# Motesanib inhibits Kit mutations associated with gastrointestinal stromal tumors

**DOI:** 10.1186/1756-9966-29-96

**Published:** 2010-07-15

**Authors:** Sean Caenepeel, Lisa Renshaw-Gegg, Angelo Baher, Tammy L Bush, Will Baron, Todd Juan, Raffi Manoukian, Andrew S Tasker, Anthony Polverino, Paul E Hughes

**Affiliations:** 1 Department of Oncology Research, Amgen Inc., One Amgen Center Drive, Thousand Oaks, CA, 91320-1799, USA; 2 Department of Protein Science, Amgen Inc., One Amgen Center Drive, Thousand Oaks, CA, 91320-1799, USA; 3 Department of Molecular Sciences, Amgen Inc., One Amgen Center Drive, Thousand Oaks, CA, 91320-1799, USA; 4 Department of Medicinal Chemistry, Amgen Inc., One Amgen Center Drive, Thousand Oaks, CA, 91320-1799, USA; 5 Department of Oncology, Amgen, Inc., 1201 Amgen Court West, Seattle, WA, 98119-3105, USA

## Abstract

**Background:**

Activating mutations in Kit receptor tyrosine kinase or the related platelet-derived growth factor receptor (PDGFR) play an important role in the pathogenesis of gastrointestinal stromal tumors (GIST).

**Methods:**

This study investigated the activity of motesanib, an inhibitor of vascular endothelial growth factor receptors (VEGFR) 1, 2, and 3; PDGFR; and Kit, against primary activating Kit mutants and mutants associated with secondary resistance to imatinib. Single- and double-mutant isoforms of Kit were evaluated for their sensitivity to motesanib or imatinib in autophosphorylation assays and in Ba/F3 cell proliferation assays.

**Results:**

Motesanib inhibited Kit autophosphorylation in CHO cell lines expressing primary activating mutations in exon 9 (AYins503-504, IC_50 _= 18 nM) and exon 11 (V560 D, IC_50 _= 5 nM; Δ552-559, IC_50 _= 1 nM). Motesanib also demonstrated activity against kinase domain mutations conferring imatinib resistance (V560D/V654A, IC_50 _= 77 nM; V560D/T670I, IC_50 _= 277 nM; Y823 D, IC_50 _= 64 nM) but failed to inhibit the imatinib-resistant D816V mutant (IC_50 _> 3000 nM). Motesanib suppressed the proliferation of Ba/F3 cells expressing Kit mutants with IC_50 _values in good agreement with those observed in the autophosphorylation assays.

**Conclusions:**

In conclusion, our data suggest that motesanib possesses inhibitory activity against primary Kit mutations and some imatinib-resistant secondary mutations.

## Background

Approximately 85% to 90% of all cases of gastrointestinal stromal tumors (GIST) are associated with gain-of-function mutations in the gene *KIT *[1-4]. A further 5% to 10% of cases of GIST are associated with activating mutations in the platelet-derived growth factor receptor alpha (PDGFRα) gene [1,4,5]. Activating Kit mutations in GIST occur principally in the extracellular domain, the juxtamembrane domain (which regulates receptor dimerization), kinase domain I, and kinase domain II (or activation loop) [1]. Imatinib, a small-molecule inhibitor of Kit and PDGFRα, represents an effective first-line therapy option for patients with advanced GIST [6]. Imatinib is a potent inhibitor of wild-type Kit and juxtamembrane domain Kit mutants, while Kit activation loop mutants are resistant [1,7]. Secondary imatinib resistance is most commonly associated with the acquisition of a secondary mutation in Kit (either in the kinase domain I or the activation loop) or in PDGFRα [8].

Motesanib is an orally administered small-molecule antagonist of vascular endothelial growth factor receptors (VEGFR) 1, 2, and 3; PDGFR and Kit [9,10]. In clinical studies, motesanib has shown encouraging efficacy in the treatment of patients with advanced solid tumors [10-13]. In biochemical assays, motesanib potently inhibits the activity of both Kit (50% inhibitory concentration [IC_50_] = 8 nM) and PDGFR (IC_50 _= 84 nM) [9], suggesting that it may have direct antitumor activity in GIST [14,15]. The aim of this study was to characterize the ability of motesanib to inhibit the activity of wild-type Kit in vitro and in vivo, and to investigate differences in the potency of motesanib and imatinib against clinically important primary activating Kit mutants and mutants associated with secondary imatinib resistance. The results suggest that motesanib has inhibitory activity against primary Kit mutations and some imatinib-resistant secondary mutations.

## Methods

### Reagents

Unless specified otherwise all reagents were purchased from Sigma Aldrich; all cell culture reagents were purchased from Invitrogen (Carlsbad, CA).

### In Vivo Hair Depigmentation Assay

Female C57B6 mice (6 to 8 weeks old; 20 to 30 g; Charles River Laboratories, Wilmington, MA) were anesthetized, and an area of skin 2 × 2 cm on the right flank was depilated. Oral administration of either 75 mg/kg motesanib (Amgen Inc., Thousand Oaks, CA) or vehicle (water, pH 2.5) was initiated on the same day as depilation and continued for 21 days. On day 21, photographs were taken for assessment of hair depigmentation. The same patch of skin was depilated again on day 28, and photographs for assessment of depigmentation were taken on day 35. All animal experimental procedures were conducted in accordance with the guidelines of the Amgen Animal Care and Use Committee and the Association for Assessment and Accreditation of Laboratory Animal Care standards.

### Preparation of Wild-Type and Mutant *KIT *Constructs

*KIT *mutants (Table 1) were identified from published reports [8] and generated using PCR-based site-directed mutagenesis. PCR products were cloned into the pcDNA3.1+ hygro vector or the pDSRα22 vector (Amgen Inc), gel purified, and then ligated with a common 5' fragment of human wild-type *KIT *to yield full-length, mutant constructs in pcDNA3.1+ hygro or pDSRα22 expression vectors.

**Table 1:** Clinically Relevant *KIT *Mutations

*KIT *Genotype	*Mutation Type*	*Domain*
Primary activating mutations		
Δ552-559	Deletion	Juxtamembrane domain
V560D	Single mutation	Juxtamembrane domain
AYins503-504	Insertion	Extracellular domain
Secondary imatinib-refractory mutations		
D816V	Single mutation	Activation loop
Y823D	Single mutation	Activation loop
V560D/V654A	Double mutation	Juxtamembrane domain/kinase domain I
V560D/T670I	Double mutation	Juxtamembrane domain/kinase domain I

### Stable Transfection of CHO and Ba/F3 Cells With Wild-Type and Mutant KIT

AM-1/D Chinese Hamster Ovary (CHO) cells (Amgen Inc.) were maintained under standard conditions. Cells were transfected with wild-type or mutant *KIT *using Lipofectamine2000 and Opti-MEM (Invitrogen) following the manufacturer's instructions. Four days after transfection, cells were transferred into selection medium: Gibco DMEM High Glucose with 10% FBS plus 300 μg/mL hygromycin (Roche Applied Sciences, Indianapolis, IN) for cells transfected with pcDNA3.1+ hygro; DMEM High Glucose with 10% dialyzed FBS for cells transfected with pDSRα22. Stably transfected CHO cells were selected 2 weeks later and maintained as described above.

Interleukin 3 (IL-3)-dependent Ba/F3 cells were maintained under standard conditions including 3 ng/mL murine IL-3 (Cat # PMC0035; Invitrogen/BioSource). Cells were transfected with wild-type or mutant *KIT *in the pDSRa22 expression vector along with linearized pcDNA Neo using the Nucleofector Kit V and a Nucleoporator (Lonza; Cologne, Germany) following the manufacturer's instructions. Two to 3 days post transfection, cells were transferred into selection medium (supplemented RPMI medium plus 750 μg/mL G418). Stably transfected Ba/F3 cells were maintained in supplemented RPMI medium plus 3 ng/mL murine IL-3.

Fluorescence activated cell sorting (FACS) was utilized to isolate pools of CHO and Ba/F3 cells stably expressing wild-type and mutant *KIT *variants. FACS was performed on a FACS Aria cell sorter (BD Biosciences San Jose, CA), under sterile conditions using 488 nm laser excitation. *KIT *transfected cells were labeled with the anti-Kit monoclonal antibody SR1 (prepared at Amgen Inc.; data on file) followed by incubation with FITC-labeled secondary anti-mouse IgG antibody (SouthernBiotech, Birmingham, AL). Cells were then resuspended in Dulbecco's phosphate-buffered saline with 0.5% bovine serum albumin at a final concentration of 1 × 10^6 ^cells per mL to ensure a constant and viable sorting rate of 5000 cells/sec. Cells transfected with vector control were used to adjust the baseline instrument settings. Forward and side scatter gating enabled the exclusion of dead cells and debris. The top 10% to 15% of Kit-positive cells within the overall transfected cell population were then isolated to ensure collection of high-expressing cells. Cells were sorted directly into 15 mL conical tubes containing the appropriate growth media. Cell pools were then cultured and maintained under the respective selection conditions, and were reanalyzed for Kit expression prior to characterization of Kit autophosphorylation.

### Cell-Based Kit Autophosphorylation Assay

CHO cells stably transfected with wild-type or mutant isoforms of *KIT *were seeded in a 96-well tissue culture plate at a density of 2 × 10^4 ^cells per well. For stem cell factor (SCF) characterization experiments, cells were stimulated with serial dilutions of SCF for varying times. To determine IC_50 _values, the cells were treated for 2 hours with single 10-fold serial dilutions of motesanib or imatinib starting at 3 μM. Cell lines transfected with wild-type *KIT *were stimulated for 10 minutes with 100 ng/mL SCF following treatment with motesanib or imatinib. Cell lines transfected with activating *KIT *mutants were not stimulated with SCF in IC_50 _experiments. Cells were washed with phosphate-buffered saline and lysed in RIPA buffer (50 mM Tris, pH 7; 150 mM NaCl, 1% Igepal, 0.5% sodium deoxycholate, 0.1% SDS, 300 μM activated sodium vanadate, 1× protease inhibitor cocktail) for 30 minutes at 4°C with shaking. Cell lysates were added to a 96-well DELFIA microplate (PerkinElmer Inc.) coated with anti-Kit antibody (1 μg per well; AF332, R&D Systems, Inc.; Minneapolis, MN) and incubated for 2 hours. Lysates were then removed and the plate was washed 3 times with DELFIA wash buffer (PerkinElmer Inc.). Recombinant anti-phosphotyrosine antibody 4G10 (Cat. # 05-777; Upstate/Millipore, Billerica, MA) was added to each well (0.1 μg per well) and incubated at room temperature for 1 hour. The plate was then washed 3 times with DELFIA wash buffer before 0.01 μg of Eu-N1-labeled anti-mouse antibody (Cat. # AD0124, PerkinElmer Inc.) was added to each well. The plate was again incubated at room temperature for 1 hour and then washed 3 times with DELFIA wash buffer before the signal was detected by adding DELFIA enhancement buffer (PerkinElmer Inc.) to each well. Luminescence was measured using a Victor Model 1420 multilabel counter (PerkinElmer Inc.). Kit autophosphorylation at each motesanib or imatinib concentration was expressed as a percentage of the vehicle control (0.2% DMSO).

### Ba/F3 Functional Viability Assay

The ability of Kit mutants to act as survival factors was assessed in Kit-dependent Ba/F3 cells. Ba/F3 cells stably transfected with various *KIT *mutants were seeded in a 96-well tissue culture plate at a density of 5 × 10^3 ^cells per well. To determine IC_50 _values, cells were treated for 24 hours with single 10-fold serial dilutions of motesanib or imatinib starting at 3 μM (0.1 μM for motesanib-treated V560 D and Δ552-559 Kit mutants). Cell viability was assessed by measuring the level of adenosine triphosphate using ATPlite assays (PerkinElmer Life Sciences, Boston, MA). Reconstituted ATPLite 1-step solution was added to each well followed by incubation with shaking for 2 minutes. The plate was read on a Victor Model 1420 multilabel counter (PerkinElmer Inc.) under the luminescence setting. Viability at each motesanib or imatinib concentration was expressed as a percentage of the vehicle control (0.2% DMSO).

## Results

### In Vitro Inhibition of Wild-Type Kit by Motesanib

Motesanib potently inhibited SCF-induced autophosphorylation of Kit in CHO cells stably transfected with the wild-type *KIT *gene (IC_50 _= 36 nM). In comparison, imatinib inhibited wild-type Kit with an IC_50 _of 165 nM.

### Inhibition of Wild-Type Kit Activity in Mice by Motesanib

**Figure 1:**
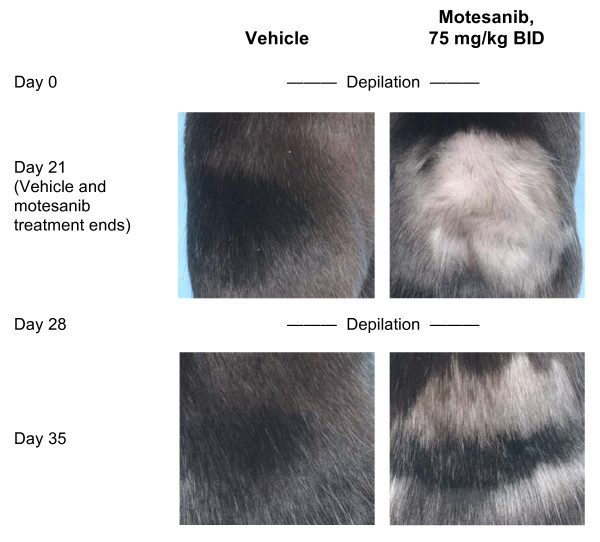
**Effect of treatment with motesanib or vehicle on hair depigmentation, a surrogate marker of Kit activity **[16], **in female C57B6 mice**. Anesthetized animals were depilated and immediately treated with either vehicle (water; left panels) or motesanib 75 mg/kg BID (right panels) for 21 days. On day 21, hair depigmentation was assessed. Depilation was repeated on day 28 and hair depigmentation was again assessed on day 35. Representative images from each treatment group for the day-21 and day-35 time points are shown. BID = twice daily.

Hair depigmentation was used as a surrogate marker to assess the ability of motesanib to inhibit Kit activity in vivo [16]. Following depilation, female C57B6 mice were administered either 75 mg/kg motesanib (n = 8) or vehicle (n = 8) twice daily for 21 days. In mice receiving motesanib, hair regrowth was markedly depigmented compared with mice receiving vehicle (Figure 1). This effect was reversible. Following the cessation of motesanib treatment on day 21, the mice were depilated again on day 28. There was no apparent depigmentation of regrown hair on day 35. Similar results were obtained in male mice (data not shown).

### Characterization of Kit Mutants

**Figure 2:**
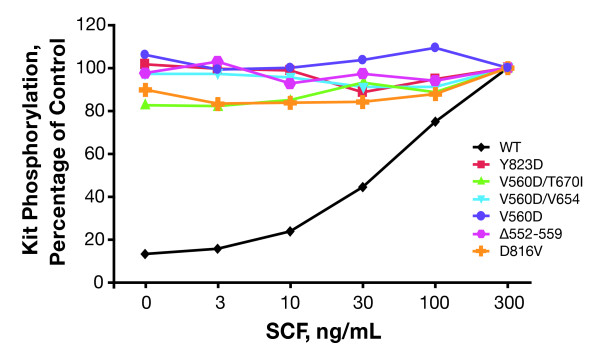
**Effect of stem cell factor (SCF) treatment on tyrosine phosphorylation of wild-type Kit and mutant Kit isoforms stably expressed in Chinese hamster ovary cells**. Chinese hamster ovary cells stably transfected with wild-type (WT) or mutant *KIT *isoforms were stimulated with single serial dilutions of stem cell factor, and Kit phosphorylation was assessed. For mutant Kit isoforms, data are expressed as the percentage of vehicle control. For wild-type Kit, data are expressed as the percentage of phosphorylation observed following stimulation with 300 ng/mL SCF. The results of a single experiment are shown.

Figure 2 summarizes the results from the autophosphorylation experiments using CHO cells stably transfected with the wild-type *KIT *gene or various *KIT *mutant genes. Tyrosine phosphorylation of wild-type Kit was dose-dependent, with the greatest intensity of autophosphorylation occurring after a 30 minute incubation of the cells with 300 ng/mL of SCF. In contrast, tyrosine phosphorylation of activated Kit mutants occurred in the absence of SCF with no further phosphorylation induced by treatment with SCF.

### Activity of Motesanib against Primary Activating Kit Mutants

**Figure 3:**
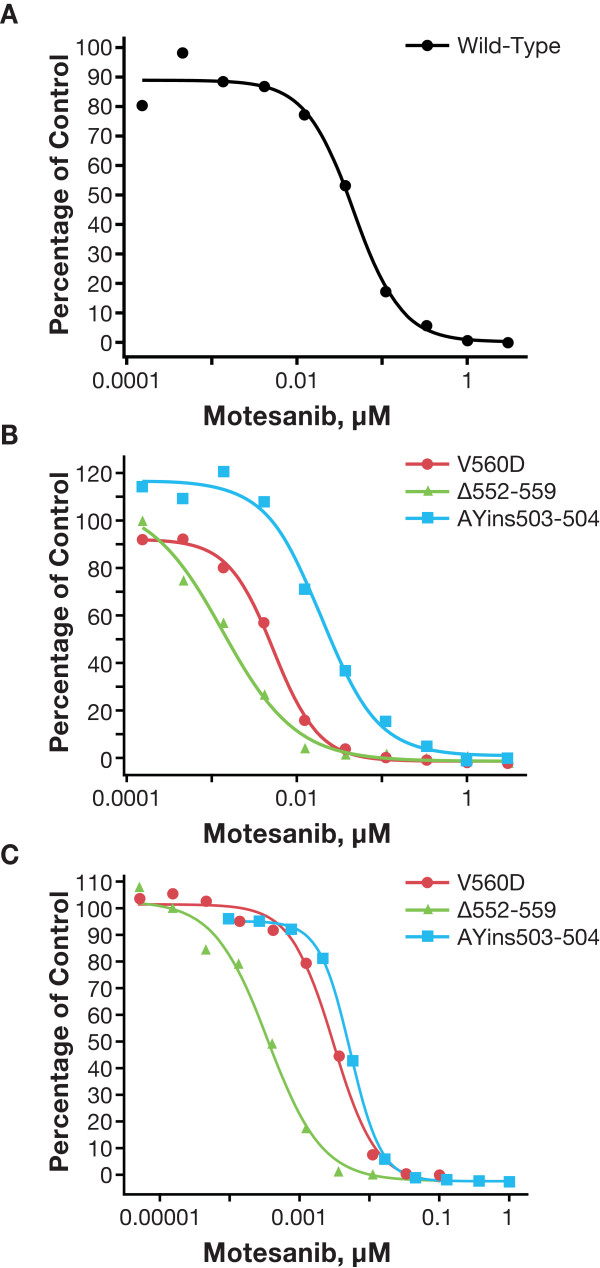
**Inhibition of the activity of wild-type Kit and primary activating Kit mutants by motesanib**. Autophosphorylation (expressed as a percentage of vehicle control) of wild-type Kit (panel A) and primary activating Kit mutants (panel B) was assessed in stably transfected Chinese hamster ovary cells treated for 2 hours with single 10-fold serial dilutions of motesanib. Representative data from 1 of 2 experiments are shown. Viability (expressed as the percentage of vehicle control) of Ba/F3 cells expressing the same primary activating Kit mutants treated for 24 hours with single 10-fold serial dilutions of motesanib was also assessed (panel C). Viability experiments were performed once (representative curves are shown).

In CHO cells, motesanib inhibited the autophosphorylation of the primary activating Kit mutants V560 D, Δ552-559, and AYins503-504 (Table 2; Figure 3B). In each instance, motesanib was a more potent inhibitor of Kit autophosphorylation than imatinib. For example, motesanib inhibited the AYins503-504 mutant with an IC_50 _of 18 nM, whereas imatinib inhibited this mutant with an IC_50 _of 84 nM. Interestingly, the IC_50 _values for inhibition of these Kit mutants were lower than the IC_50 _for inhibition of wild-type Kit by motesanib. Consistent results were obtained in a functional viability assay utilizing IL-3-independent growth of Ba/F3 cells (Figure 3C). For example, when testing the AYins503-504 mutant, the IC_50 _for motesanib was 11 nM versus 47 nM for imatinib.

**Table 2:** Inhibition of the Activity of Wild-Type Kit and Primary Activating Kit Mutants by Motesanib and Imatinib*

	IC_50 _of Kit Autophosphorylation, nM		IC_50 _of Stably Transfected Ba/F3 Cell Survival, nM	
		
*KIT *Genotype	Motesanib	Imatinib	Motesanib	Imatinib
Wild-type	36	165	-	-
V560D	5	18	3	7
Δ552-559	1	5	0.4	1
AYins503-504	18	84	11	47

*In autophosphorylation experiments, means from 2 experiments are shown, with the exception of Δ552-559, which was assessed once. Viability experiments were performed once.

### Activity of Motesanib against Imatinib-Resistant Kit Mutants

**Figure 4:**
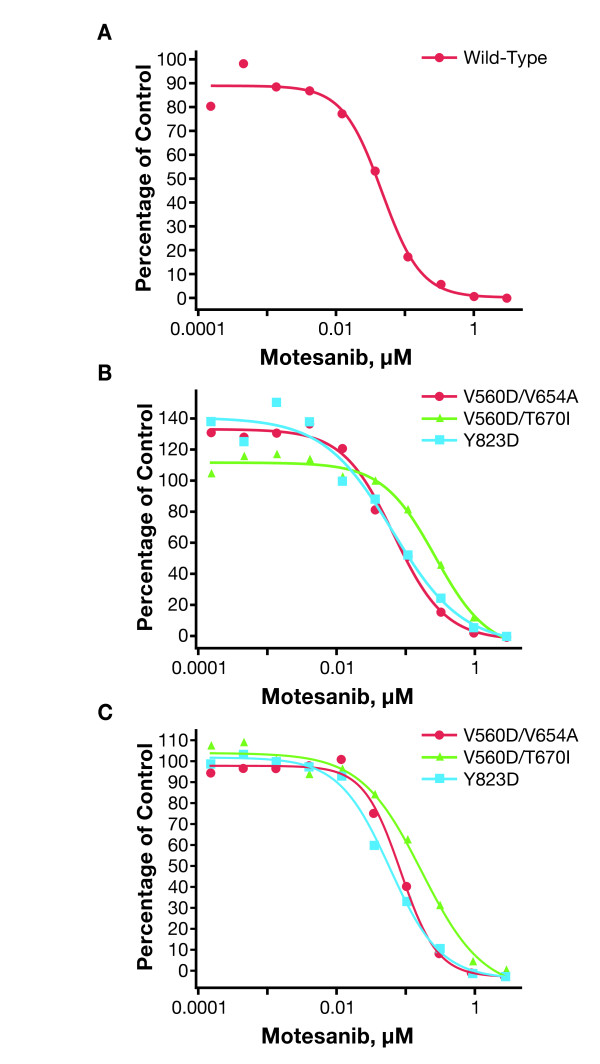
**Inhibition of the activity of Kit mutants associated with secondary imatinib resistance by motesanib**. Autophosphorylation (expressed as a percentage of vehicle control) of wild-type Kit (panel A) and Kit mutants associated with secondary imatinib resistance (panel B) was assessed in stably transfected Chinese hamster ovary cells treated for 2 hours with single 10-fold serial dilutions of motesanib. Representative data from 1 of 2 experiments are shown. Viability (expressed as the percentage of vehicle control) of Ba/F3 cells expressing the same Kit mutants treated for 24 hours with single 10-fold serial dilutions of motesanib was also assessed (panel C; not shown: D816V, which had a motesanib IC_50 _> 3 μM). Viability experiments were performed once and representative curves are shown (D816V was not evaluated because Ba/F3 cells expressing this mutant could not be established).

Motesanib inhibited the activity of Kit mutants associated with secondary imatinib resistance. In Kit autophosphorylation assays, motesanib inhibited tyrosine phosphorylation of the juxtamembrane domain/kinase domain I double mutants V560D/V654A and V560D/T670I with IC_50 _values of 77 nM and 277 nM, respectively. Imatinib had limited activity against the V560D/V654A mutant and no activity against the V560D/T670I mutant at concentrations of up to 3000 nM (Table 3; Figure 4B). Consistent results were obtained in the Ba/F3 cells expressing the V560D/V654A and V560D/T670I mutants with motesanib IC_50 _values of 91 nM and 180 nM, respectively. Again, motesanib was a more potent inhibitor of these mutants than imatinib (Table 3; Figure 4C).

**Table 3:** Inhibition of the Activity of Kit Mutants Associated With Imatinib Resistance by Motesanib and Imatinib*

	IC_50 _of Kit Autophosphorylation, nM		IC_50 _of Stably Transfected Ba/F3 Cell Survival, nM	
		
*KIT *Genotype	Motesanib	Imatinib	Motesanib	Imatinib
V560D/V654A	77	319	91	145
V560D/T670I	277	>3000	180	>3000
Y823D	64	>3000	62	330
D816V	>3000	>3000	-	-

*In autophosphorylation experiments, means from 2 experiments are shown, with the exception of V560D/V654A and D816V, which were assessed once. Viability experiments were performed once.

Similarly, motesanib inhibited autophosphorylation of the imatinib-resistant activation loop mutant Y823 D (IC_50 _= 64 nM) more potently than imatinib (IC_50 _> 3000 nM) (Table 3: Figure 4B). However, neither motesanib nor imatinib inhibited autophosphorylation of the D816V mutant (Table 3). Consistent with these results, motesanib inhibited the growth of Ba/F3 cells transfected with the V560D/V654A, V560D/T670I, or Y823 D mutant more potently than imatinib. Of note, the IC_50 _of imatinib against the Y823 D mutant when established in the functional viability assay was at least 10-fold lower than the IC_50 _measured in the autophosphorylation assay. IL-3-independent Ba/F3 cells expressing the D816V Kit mutant could not be established.

## Discussion

In this study, motesanib was found to be a potent inhibitor of wild-type Kit, both in vitro and in vivo. In a surrogate marker assay, we observed reversible hair depigmentation in mice treated with motesanib 75 mg/kg twice daily. This dose is comparable to the doses used in xenograft studies demonstrating antitumor and antiangiogenic properties of motesanib [9,17]. Kit signaling plays an important role in the regulation of hair follicle melanocytes, likely through control of tyrosinase and tyrosinase-related protein 1 (TRP1) expression [16]. Depigmentation has previously been observed in mice treated with anti-Kit antibodies [16,18] or with sunitinib [18]. Importantly, motesanib had inhibitory activity against Kit mutants associated with GIST and inhibited these mutants more potently than imatinib and generally with an IC_50 _that was less than or similar to the 24-hour trough concentration of motesanib at therapeutic doses in humans [10].

Motesanib was a more potent inhibitor of the primary activating juxtamembrane domain and extracellular domain Kit mutants V560 D, Δ552-559, and AYins503-504, compared with imatinib. Importantly, motesanib also inhibited the activity of an activation loop mutant (Y823D) associated with imatinib resistance. Imatinib did not inhibit this mutant at concentrations of up to 3000 nM, suggesting that there are marked differences in how the two inhibitors interact with Kit. We previously solved the structure of motesanib bound to the VEGFR2 kinase domain at 2.2 Å resolution (PDB Accession Code 3EFL) [19]. This structure superimposes favorably with that of Kit co-crystallized with imatinib (PDB Accession Code 1T46) [20]. Both inhibitors bind the inactive, auto-inhibited form of the kinases with the backbone of the protein reorganized into the so-called "DFG-out" conformation. Based on the structural similarities and the similar potencies of motesanib against VEGFR2 and Kit, we reasoned that motesanib binds these target kinases in exactly the same fashion.

**Figure 5:**
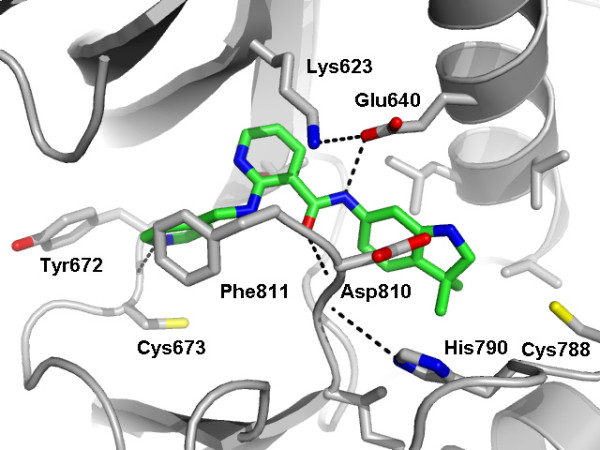
A model of motesanib bound to the active site of Kit kinase derived from a 2.2 Ångstrom resolution crystal structure of motesanib bound to the active site of VEGFR2 kinase (PDB code 2EFL).

Modeling studies suggest that motesanib engages Kit via three polar interactions and a multitude of van der Waals contacts (Figure 5). In the context of this study, the most important of these interactions are those with threonine 670 via a non-classical CH-O pseudo hydrogen bond and interactions with valine 654 through hydrophobic contacts. The fifteen-fold loss of motesanib activity (5 nM versus 77 nM) noted with the V560D/V654A double mutant, compared with V560 D alone, is rationalized by the loss of two van der Waals contacts with alanine 654 in a similar fashion to that described for imatinib [21,22].

Motesanib and imatinib have much diminished activity against the activation loop mutant (D816V). The D816V mutant destabilizes the inactivated form of Kit, in a way that the ability of the protein to adopt the "DFG out" (inactive) conformation is much reduced or even eliminated; thus, the mutation prevents both motesanib and imatinib from binding to the ATP pocket [23,24]. The failure to potently inhibit the D816V mutation is a feature of Kit inhibitors in the clinic, with the exception for dasatinib [23,25,26], which binds the "DFG in", or activated form, of the kinase [27]. However, the ability of motesanib to inhibit the Y823 D mutant suggests that its activity may not be entirely restricted to an inactive protein conformation, or alternatively it may reflect that in contrast to the D816V mutation, the conformational equilibrium of the Y823 D mutant is not shifted permanently to the active conformation.

The data from the present study are of translational relevance, supporting evidence indicating that targeted therapy molecules with different binding sites and/or mode of action may be required in the treatment of cancers for which mutations are the primary oncogenic event. A recent study has demonstrated that differences in the conformational structure of Kit mutants influences the ability of sunitinib [28], and imatinib to bind and inhibit receptor autophosphorylation, thus providing a unique mechanism of drug resistance for each mutant that is unlikely to be overcome using a single treatment [23].

## Conclusions

In summary, the results of this study demonstrate that different Kit mutations respond differently to motesanib or imatinib. This likely reflects differences in the molecules' mode of action. The data also show that motesanib is active against Kit mutations associated with resistance, suggesting that it may have clinical utility in the treatment of patients with primary and secondary imatinib-resistant GIST.

## Competing interests

All authors are employees of and shareholders in Amgen Inc.

## Authors' contributions

SC designed the cell viability and Kit autophosphorylation assays. LRG contributed to the generation of cell lines expressing wild-type and mutant Kit. AB performed the depilation experiments. TLB performed the depilation experiments. WB designed and generated wild-type and mutant *KIT *gene expression vectors. TJ designed and generated wild-type and mutant *KIT *gene expression vectors. RM contributed to the generation of cell lines expressing wild-type and mutant Kit. AST contributed the molecular modelling and assisted with the writing of the manuscript. AP was responsible for the overall experimental design and contributed to the writing of the manuscript. PEH was responsible for individual experimental designs and contributed to the writing of the mansucript. All authors have read and approved the final manuscript.
